# Structural and mechanistic characterization of an archaeal-like chaperonin from a thermophilic bacterium

**DOI:** 10.1038/s41467-017-00980-z

**Published:** 2017-10-10

**Authors:** Young Jun An, Sara E. Rowland, Jung-Hyun Na, Dario Spigolon, Seung Kon Hong, Yeo Joon Yoon, Jung-Hyun Lee, Frank T. Robb, Sun-Shin Cha

**Affiliations:** 1 Marine Biotechnology Research Center, Korea Institute of Ocean Science and Technology, Ansan, 15627 Republic of Korea; 2 Department of Microbiology and Immunology, University of Maryland, Baltimore, MD 21201 USA; 3 Institute of Marine and Environmental Technology, Baltimore, MD 21201 USA; 4 Department of Chemistry & Nanoscience, Ewha Womans University, Seoul, 03760 Republic of Korea

## Abstract

The chaperonins (CPNs) are megadalton sized hollow complexes with two cavities that open and close to encapsulate non-native proteins. CPNs are assigned to two sequence-related groups that have distinct allosteric mechanisms. In Group I CPNs a detachable co-chaperone, GroES, closes the chambers whereas in Group II a built-in lid closes the chambers. Group I CPNs have a bacterial ancestry, whereas Group II CPNs are archaeal in origin. Here we describe open and closed crystal structures representing a new phylogenetic branch of CPNs. These Group III CPNs are divergent in sequence and structure from extant CPNs, but are closed by a built-in lid like Group II CPNs. A nucleotide-sensing loop, present in both Group I and Group II CPNs, is notably absent. We identified inter-ring pivot joints that articulate during ring closure. These Group III CPNs likely represent a relic from the ancestral CPN that formed distinct bacterial and archaeal branches.

## Abstract (web summary)

Chaperonins (CPNs) are ATP-dependent protein-folding machines. Here the authors present the open and closed crystal structures of a Group III CPN from the thermophilic bacterium *Carboxydothermus hydrogenoformans*, discuss its mechanism and structurally compare it with Group I and II CPNs.

## Introduction

Molecular chaperones have an essential role in ensuring proper folding of cellular proteins. Two ATP-dependent protein-folding machines, Hsp70 (DnaK) and the chaperonin (CPN) are present in the majority of cells^1–7^. Both systems fold nascent polypeptides as well as salvage and recycle stress-denatured proteins^5, 8–10^. While the CPNs or Hsp70 complexes are absent in some species, this is rare and there are no reports of an organism lacking both complexes. The CPN of *Escherichia coli*, GroEL-GroES, is the most intensely studied molecular chaperone. Each CPN ring encloses a large solvent-filled cavity and the cavity-facing surface of the open ring is lined with hydrophobic amino acids that capture incompletely folded substrate proteins. Binding and hydrolysis of ATP result in allosteric conformational changes of the CPN complex, coupling ring closure with encapsulation of non-native client proteins inside their chambers. Based on the disparity in structure and sequence, CPNs were previously divided into two paralogous groups^11^. Group I CPNs, with the bacterial chaperonin model GroEL/ES^12–14^, exist in bacteria, chloroplasts, and mitochondria, whereas archaeal thermosomes^15^ and CCTs (chaperonin containing TCP1) are present in the eukaryotic cytosol^16, 17^ and comprise Group II^16, 18, 19^.

Group I and Group II CPNs are clearly descended from a common CPN ancestor, since each CPN subunit is ~60 kDa with domain architecture that is shared across groups. The functional quaternary structure of both Group I and II CPNs is formed by a toroidal arrangement of subunits in a hollow double ringed complex forming two cavities that enclose non-native proteins, and individual subunits consist of an ATP-binding equatorial domain also involved in inter-ring contact, an apical substrate binding domain, and an intermediate domain linking the two domains^20, 21^. These sequence-related groups, however, differ profoundly in the mechanism of ring closure: Group I employs an independent co-chaperone called GroES^22^ as a detachable lid that binds to the apical domains of a ring, whereas in Group II CPNs long protruding helical motifs of the apical domains serve as a built-in lid to each ring^20^.

Recently, a Group II-like CPN was annotated in multiple bacterial genomes and was described as an “archaeal-like” CPN^23, 24^. Currently, there is a monophyletic clade of 195 CPN paralogs that are phylogenetically distinct from both Group I and II CPNs, thus representing a third monophyletic CPN group (Supplementary Fig. 1 and Supplementary Table 1). Group III CPNs, with one exception, are encoded within the bacterial Hsp70 (DnaK) operon^23^ suggesting that they may have a unique functional association with the bacterial DnaK/DnaJ complex.

Intense investigation of Group I, and to a lesser extent Group II CPNs, have revealed the conformational articulation accompanying opening, closing and client protein binding, through the application of crystallography^25, 26^, cryo-EM, and disulfide cross-linking^27^. Here we describe the prototype crystal structures of the bacterial Group III CPN from *Carboxydothermus hydrogenoformans* (Ch-CPN), an extremely thermophilic, carbon monoxide utilizing bacterium isolated from a hot spring in Eastern Russia. Here we present the open and closed conformations (Table 1), and the mechanistic details of ring closing and protein folding. Evolutionary affiliations among Group I, II, and III CPNs are discussed in light of the unique subunit articulation and allostery of ATP binding and hydrolysis of Ch-CPN^28^.

**Table 1:** Data collection and refinement statistics (molecular replacement)

	Ch-CPN/AMPPNP (Closed)	Ch-CPN/ADP (Open)
*Data collection*		
Space group	*P*422	*P*42_1_2
Cell dimensions		
*a*, *b*, *c* (Å)	186.17, 186.17, 160.74	209.78, 209.78, 169.81
*α*, *β*, *γ* (°)	90, 90, 90	90, 90, 90
Resolution (Å)	50.00–3.0 (3.05–3.0)	50–4.0 (4.14–4.0)
*R*_merge_	0.120 (0.414)	0.497 (2.761)
*I*/σ*I*	24.7 (5.4)	8.4 (1.0)
Completeness (%)	99.1 (98.8)	99.8 (98.3)
Redundancy	14.9 (8.8)	20.3 (18.4)
*Refinement*		
Resolution (Å)	45.15–3.0	49.45–4.0
No. reflections	56,286	32,468
*R*_work_/*R*_free_	19.85 (25.82)	25.29 (32.10)
No. atoms		
Protein	14,652	14,665
ADP		108
AMPPNP	124	
Magnesium	4	
*B*-factors		
Protein	34.97	131.33
ADP		101.36
AMPPNP	36.69	
Magnesium	33.94	
R.m.s. deviations		
Bond lengths (Å)	0.015	0.005
Bond angles (°)	1.220	1.029

Each structure was determined from a single crystal. Highest-resolution shell is shown in parentheses

## Results

### Subunit structure of Group III CPNs

**Fig. 1:**
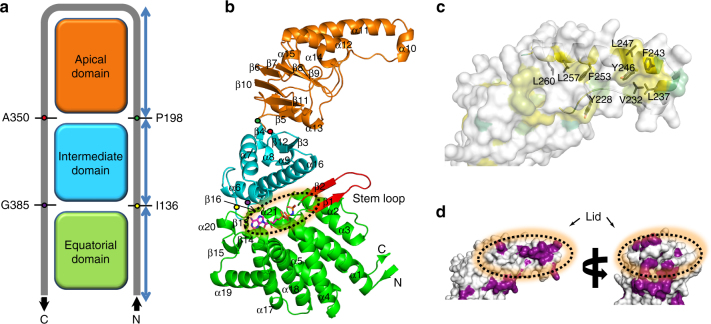
Subunit structure of Ch-CPN. Equatorial, intermediate, and apical domain are colored in green, cyan, and orange, respectively. The N- and C-termini are labeled as N and C, respectively. **a** Schematic representation of Cα tracing of Ch-CPN subunit. Four key residues connecting the domains are labeled. **b** Ribbon diagram of Ch-CPN subunit. The nucleotide-binding site and stem loop are indicated by a dotted circle and red ribbon, respectively. The key residues (I136, P198, A350, and G385) are marked by yellow, green, red, and purple circles, respectively. α-Helices and β-strands are numbered consecutively from the N-terminus to the C‐terminus. **c** Hydrophobic surface of the apical domain. The surfaces are colored based on the hydrophobicity of the side chains (from yellow for most hydrophobic, to lime for decreasing hydrophobicity). The crowded hydrophobic residues on the lid are labeled and presented as black sticks. **d** Residue conservation in the apical domain. Highly conserved residues are colored in purple. Amino acid sequences of the Group III CPNs which is used for residue conservation are described in Supplementary Fig. 2. The lid is indicated by a dotted circle

The subunits of Ch-CPN assume the modular structure consisting of an equatorial domain, an intermediate domain, and an apical domain (Figs. 1a, b). The topology of the Cα tracing shows an inverted U-shaped curve (Fig. 1a), indicating that the equatorial and intermediate domains are assembled from two segments distant from each other in the polypeptide chain whereas the apical domain is built from contiguous residues. This “inverted U” topology has been thoroughly established in Group I and Group II CPNs and is now confirmed in Group III CPNs, strongly supporting the concept that the CPNs are descended from a common ancestor. The equatorial domain of Ch*-*CPN consisting of ten α-helices and three two-stranded antiparallel β-sheets is the fusion of the N-terminal residues 1-136 and the C-terminal residues 385–502. AMPPNP and ADP are situated in a nest-like depression on top of this domain. As described below, the β1–β2 sheet that is referred to as the stem loop in CPNs is the region that senses the nucleotide state and triggers the allosteric wave of structural rearrangement during ring closure (Fig. 1b). Asp56 in this domain and Asp373 in the intermediate domain are conserved catalytic residues involved in ATP hydrolysis (Supplementary Fig. 2).

Residues 137–198 and 350–384 form the intermediate domain with a three-stranded antiparallel sheet on top of a four-helix bundle (Fig. 1a). Two helices (α6 and α16) in the helical part cover the nucleotide-binding site like a cap and a three-stranded sheet faces the apical domain. Two key residues involved in ATP hydrolysis (Arg155 between α6 and α7 and Asp373 in α16) are located in this domain (Supplementary Fig. 2). The apical domain consists of residues 199–349. The lower region mainly composed of β-strands faces the intermediate domain and the upper helical region harbors the built-in lid (α10 and α11) (Fig. 1b). Hydrophobic residues including Leu237, Phe243, Tyr246, and Phe253 that are strictly conserved in Group III CPNs crowd at the tip of the built-in lid, and appear to be involved in the binding of denatured substrate proteins or docking with co-chaperones (Figs. 1c, d, and Supplementary Fig. 2).

### The closed conformation of Ch-CPN

**Fig. 2:**
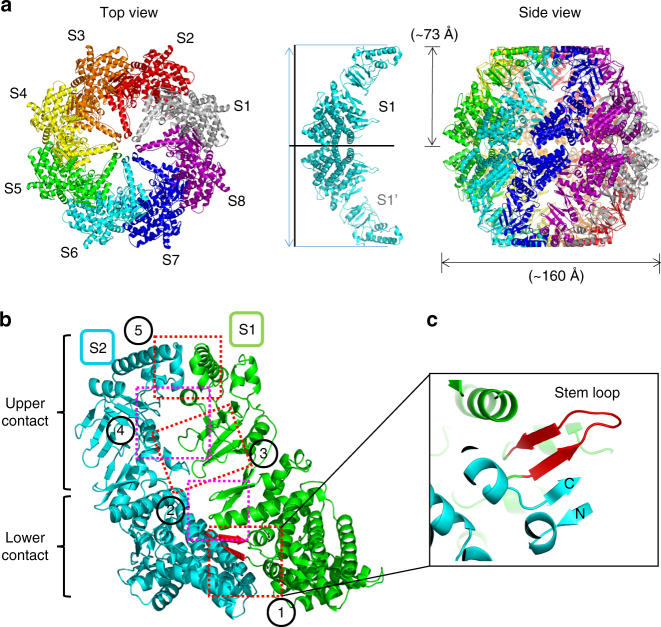
Crystal structure of Ch-CPN in the closed state. **a** Overall structure of the AMPPNP-bound Ch-CPN. Subunits are labeled as S1–S8 and shown in different colors. The conformation of an inter-ring dimer (S1 and S1′) is depicted to calculate the molecular dimension (middle). **b** Contact areas in the intra ring dimer (S1 and S2). Interactions of adjacent subunits are indicated by dotted boxes. **c** Close-up view of the inter-subunit β-sheet in the closed state. Details of other interactions (from 2 to 5) are included in Supplementary Fig. 3

The crystal structure of Ch-CPN in complex with AMPPNP, an ATP analog, revealed its closed conformation. Eight subunits (from S1 to S8) assemble into a hemisphere-like complex with ~ 73 Å in height and ~ 160 Å in diameter (Fig. 2a). Because interfaces between subunits are identical, we describe only one interface between S1 and S2 (Fig. 2b). The subunit interface can be divided into two areas: a lower and an upper contact areas. In the lower contact area, the equatorial and intermediate domains of S1 pack against the equatorial domain of S2 while the apical domain of S1 makes contacts with the intermediate and apical domains of S2 in the upper contact area (Fig. 2b).

Two inter-subunit contacts are observed in the lower contact area (Fig. 2b and Supplementary Fig. 3). First, an inter-subunit β-sheet is constructed by the stem loop in the S1 equatorial domain and the N-/C-terminal strands in the S2 equatorial domain (Fig. 2c). Both faces of the sheet sustain hydrophobic interactions with S1 and S2 equatorial domains. The second contact is the packing of the β12–α16 loop (residues 358-361) in the S1 intermediate domain against α3 and α21 in the S2 equatorial domain to form two polar interactions (Supplementary Fig. 3): Glu362 and the backbone NH of Ala359 are hydrogen-bonded to Asn76 and Glu490, respectively.

There are three major contacts in the upper contact area (Fig. 2b and Supplementary Fig. 3). First, one edge of a four-stranded antiparallel β-sheet (β5–β6–β10–β11) in the S1 apical domain interacts with the α8–α9 loop in the S2 intermediate domain with one hydrogen bond between Ser212 and Asp177 (Supplementary Fig. 3). Second, α12 and Trp208 in the S1 apical domain make interactions with the α13–α14 region in the S2 apical domain (Supplementary Fig. 3). The third contact is the hydrophobic association of two built-in lids through the tip regions with hydrophobic residues (Supplementary Fig. 3).

Two octameric hemispheres are stacked in a back-to-back manner to form a hexadecameric sphere with apical domains gathering around the two poles and equatorial domains at the equator. The stacking is mediated only by equatorial domains in such a way that each equatorial domain in one hemisphere has only one contacting partner - the S1 equatorial domain in one hemisphere directly interacts only with the S1′ equatorial domain in the opposite hemisphere. There are two contacts that are duplicated by an inherent 2-fold symmetry at the S1–S1′ interface (Supplementary Fig. 4). At both edges of the interface, Leu439 fits into a groove formed by the backbone of residues 109–111 and the side chains of Lys109, Val111, Met411, and Tyr414 (Supplementary Fig. 4). Another contact is an ion pair between Lys109 and Glu102 near the 2-fold axis (Supplementary Fig. 4).

AMPPNP binds to the interface between the equatorial and intermediate domains (Fig. 1b and Supplementary Fig. 5a). The adenine is surrounded by Pro37, Ile152, Phe461, Met469, and Val474 and the ribose ring is packed against Met430 with its OH2 hydrogen bonding with the backbone NH of Gly390. Three phosphates make multiple interactions with the nucleotide-binding site. The α-phosphate is in hydrogen bond distances from the backbone oxygen of Ser34 and Asn55. The β- and γ-phosphates are hydrogen bonded to Asp87, Thr89, and Thr91 in a conserved P-loop motif characterized by the sequence GDGTTT^29^. A magnesium ion whose source seems to be the mother liquor is coordinated by the three phosphates and Asp87, which contributes to neutralization of the negative charge of phosphates (Supplementary Fig. 5a). The γ-phosphate is further stabilized by Arg155 (Supplementary Fig. 5a) which is conserved in Group III CPNs^30^ (Supplementary Fig. 6a).

The ATPase activities of Ch-CPN wild type (WT) and R155K at 65 °C are comparable, whereas the R155E and R155L mutants have severely diminished activity (Supplementary Fig. 6b). These data indicate that Arg155 or a basic residue at this position is involved in ATP hydrolysis. It has been proposed that a water molecule activated by two acidic residues corresponding to Asp56 and Asp373 in Ch-CPN attacks the phosphorus atom of the γ-phosphate to cleave the β-γ phosphoanhydride bond of ATP^31^. Consistently, Asp56 and Asp373 are located near the γ-phosphate (Supplementary Fig. 5a). Thermostability of the D373A mutant was also compromised as it retained only 4.7% of the basal ATPase activity of Ch-CPN WT after 1 h incubation at 65 °C, while WT was stimulated 6-fold compared to 25 °C (Supplementary Fig. 6b), verifying the involvement of this residue in ATP hydrolysis.

### The open conformation of Ch-CPN

**Fig. 3:**
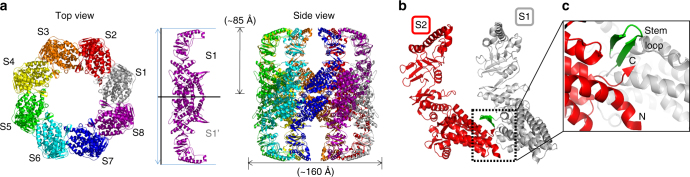
Crystal structure of Ch-CPN in the open state. **a** Overall structure of the ADP-bound Ch-CPN. Subunits are labeled as S1–S8 and shown in different colors. The conformation of an inter ring dimer (S1 and S1′) is depicted to calculate the molecular dimension (middle). **b** A contact area in the intra ring dimer is indicated by a dotted box. **c** Close-up view of the intra ring contact

**Fig. 4:**
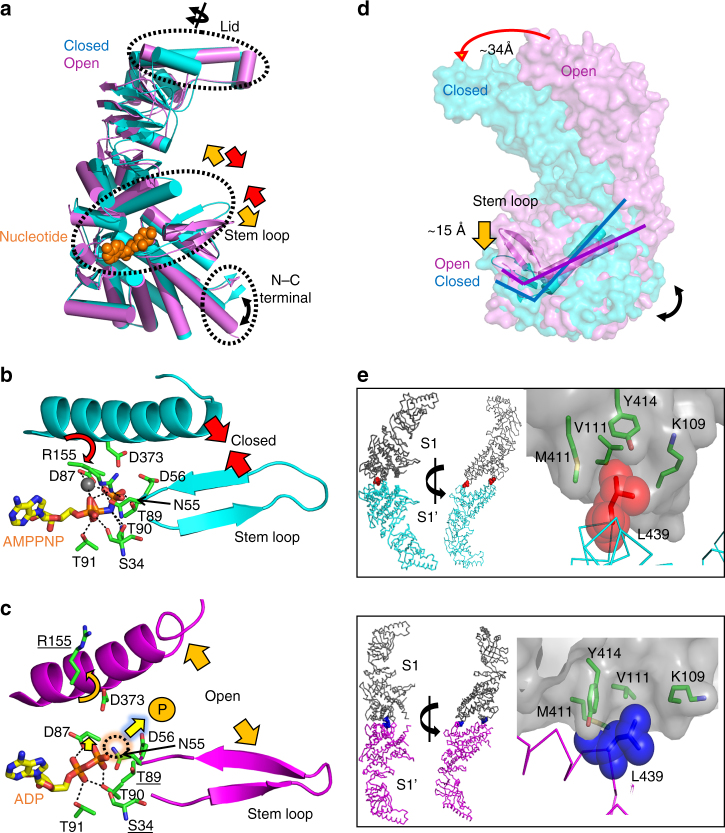
Mechanism of ring closure in Ch-CPN. **a** Superposition of the open and closed subunits. Major conformational changes are indicated by black dotted circles. **b**, **c** Movements of the stem loop and the nucleotide-sensing loop by **b** AMPPNP (cyan) or **c** ADP binding (purple). Residues interacting with nucleotides and a magnesium ion are shown in green sticks and a gray sphere, respectively. A black dotted circle indicates the position of the hydrolyzed γ-phosphate represented by P. Movements of the stem loop and the nucleotide-sensing loop are represented by red (in **b**) and orange (in **c**) arrows. Residues that are involved in interactions with AMPPNP but do not interact with ADP are indicated in bold underline letters. **d** Subunit movements on ring closure. On ATP binding, the stem loop is shifted down about 15 Å and concurrently the apical domain moves inwards about 34 Å. **e** C- and O-interfaces of the closed (upper) and open Ch-CPNs (bottom). Two Leu439 residues in the interface between S1 and S1′ mediate inter ring communication (ball and stick) in the closed state (red) and the open state (blue), respectively. Residues forming the socket are presented as sticks

The crystal structure of the ADP-bound Ch-CPN shows its fully open conformation. Eight subunits are combined to form a short cylinder with inter-subunit interactions mediated only by the equatorial domains (Fig. 3a). There is only one contact between equatorial domains of S1 and S2 (Fig. 3b). The stem loop of the S1 equatorial domain is associated with the C-terminal strand of the S2 equatorial domain to form an inter-subunit β-sheet, which contrasts with the corresponding four-stranded inter-subunit β-sheet in the closed Ch-CPN (Figs. 2c and 3c). The N-terminal residues 9–13, a β-strand in the closed Ch-CPN, assume a helical conformation in the open Ch-CPN (Figs. 3c and 4a), explaining the disparity in the number of strands between the closed and open states.

Two short cylinders are stacked to form a long cylinder with ~ 85 Å in height and ~ 160 Å in width (Fig. 3a). The stacking occurs through equatorial domains in the same 1:1 manner as observed in the closed conformation. The loop between α17 and α18 of one equatorial domain makes contacts with α19 of another equatorial domain at both edges of the S1–S1′ interface (Fig. 3a). There are hydrophobic contacts between two methionine residues (Met417) and ion pairs between Lys405 and Glu402 at the central region of the interface (Supplementary Fig. 7).

Although ADP is bound to the AMPPNP binding site, it lacks interactions with Ile152, Arg155, and Asp373 of the intermediate domain, and Asn55 and Asp56 in the stem loop compared to AMPPNP (Supplementary Fig. 5). In addition, no magnesium ion is observed despite the presence of MgCl_2_ in the mother liquor (Supplementary Fig. 5). This, together with the distant location of the intermediate domain and the stem loop from ADP, explains the decrease in interactions with ADP. It should be noted that most of the lost interactions are related to the absence of the γ-phosphate. Although the binding mode of the adenosine of ADP is nearly identical to that of AMPPNP, the diphosphate moiety of ADP assumes a different conformation, which in turn gives rise to different interactions (Supplementary Fig. 5). The α- and β-phosphates of ADP interact with Asp87, Thr89, and Thr90 in the P-loop motif whereas the α-phosphate of AMPPNP has no contact with the P-loop motif and its β-phosphate interacts with Thr91 of the P-loop motif (Supplementary Fig. 5). Overall, the loose binding of ADP, the product of ATP hydrolysis, facilitates the product (ADP) release to vacate the site during ATP binding, initiating a new cycle of protein folding activity.

### Overall comparison between Group II and III CPNs

**Fig. 5:**
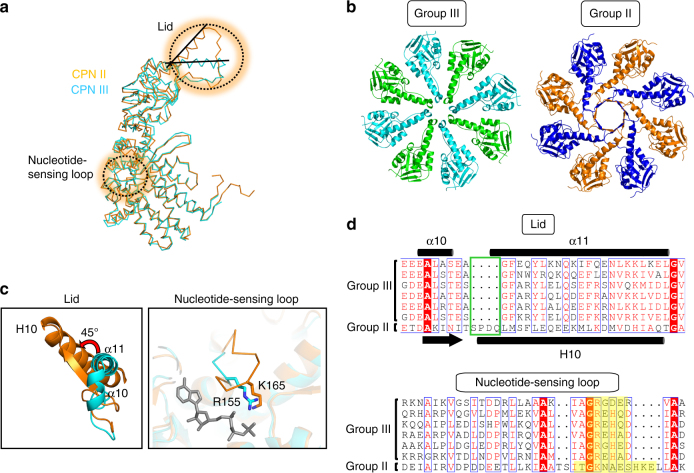
Comparison of Ch-CPN with Group II CPN. **a** Superposition of subunit structures of Ch-CPN (cyan) and Group II TKS1-CPN (PDB entry: 1Q3S^31^, orange). Significantly different regions between Group II and III CPNs are indicated by dotted circles. **b** Top views of the lid of Group II (right) and Group III CPNs (left). **c** Close-up views of the lid (left) and nucleotide-sensing loop (right) regions of Group II and III CPNs. The lid of TKS1-CPN (orange) is rotated 45° relative to Ch-CPN (cyan). In the nucleotide-sensing loop, Arg155 of Group III CPN (cyan) is equivalent to Lys165 (orange) in Group II. Bound AMPPNP is shown in a gray stick. **d** Structure-based sequence alignments of the lid (upper) and nucleotide-sensing loop (bottom) in Group II and Group III CPNs. Deletions in the lid of Group III CPNs are indicated by a green box (upper). Residues constituting nucleotide-sensing loops are highlighted in yellow backgrounds (bottom)

In structural aspects, Ch-CPN resembles archaeal Group II more than bacterial Group I CPNs (Supplementary Fig. 8 and 9). The latter are tetradecamers, have co-chaperones for ring closure, and show staggered subunit arrangements at the inter-ring interface. Like Group II CPNs, Ch-CPN is a hexadecamer consisting of a doublet of octameric rings, its subunits interact between the rings in a direct rather than staggered subunit to subunit fashion, and achieve cavity closure independent of co-chaperones. The open and closed structures of Ch-CPN seem to be similar to those of *Thermococcus* strain KS-1 CPN (TKS1-CPN) (Fig. 5a)^31^, an archaeal Group II CPN for structural comparison. Consistently, the subunit structures of Ch-CPN and TKS1-CPN are superposed with root-mean-square deviations of  ~ 1.64 Å for 440 corresponding Cα atoms in spite of their low sequence identity (26.1%). However, there are notable differences between Ch-CPN and TKS1-CPN. In their closed states, the contact surface area at the ring-ring interface in Ch-CPN is 4700 Å^2^ (3.2% of total surface area) whereas that in TKS1-CPN is 6600 Å^2^ (4.5% of total surface area). The volume of the folding chambers in Ch-CPN is 463,648 Å^3^, which is intermediate between Group I (605,512 Å^3^) and Group II CPN (382,000 Å^3^) (Supplementary Fig. 8).

Furthermore, the surface electrostatic potential of the folding chamber in Ch-CPN are different from those in TKS1-CPN (Supplementary Fig. 9). For clarity and comparison, the surface of the folding chambers in Ch-CPN and TKS1-CPN can be divided into three layers. The first layer represents the lid region, the second layer is a positively charged belt, and the third layer refers to the remaining region below the second layer. The electrostatic characteristics of the folding chambers are different between these two CPNs (Supplementary Fig. 9). The first layer of Ch-CPN contains a negatively charged hole whereas the corresponding hole of TKS1-CPN is positively charged. In the case of the second layer, the feature of the positive electrostatic potential is more obvious in TKS1-CPN than in Ch-CPN. Moreover, the third layer of Ch-CPN contains both positively and negatively charged parts, which contrasts with the overwhelming negative electrostatic potential in the corresponding layer of TKS1-CPN.

### Mechanism of chamber closing in Ch-CPN

The closed state with a non-hydrolyzable ATP analog and the open state with ADP represent the conformational states of Ch-CPN before and after ATP hydrolysis, respectively. The open form is more accessible to digestion by proteinase K as shown by the results of running digests of the complex with ADP, ATP, and AMPPNP (Supplementary Fig. 10). The simplest interpretation of this result is that the open ADP-bound Ch-CPN is sensitive to proteinase K whereas the closed ATP- or AMPPNP-bound chaperonins are relatively protease-resistant forms, indicating that the binding of AMPPNP to Ch-CPN induces the closed conformation.

To visualize the conformational changes before and after ATP binding at the subunit level, the structure of a subunit from the open conformation (O-subunit) is superposed onto that of a subunit from the closed conformation (C-subunit) with equatorial domains as reference (Fig. 4a). Although the equatorial domains are well matched, there are two remarkable structural differences between the open and closed subunits. One difference is directly related to the distinct nucleotide states of the two subunits. Asn55 and Asp56 in the so-called stem loop, and Arg155 and Asp373 in the intermediate domain interact with the α- and γ-phosphates of AMPPNP in C-subunit (Fig. 4b). However, the four residues are disengaged from the di-phosphates of ADP in O-subunit (Fig. 4c). Consequently, the intermediate domain and the stem loop harboring the four residues are close to each other in C-subunit whereas they are distantly located in O-subunit. This observation suggests a conformational change of subunits during the open to closed transition: on ATP-binding to O-subunit, the intermediate domain and the stem loop move toward ATP to locate the four residues at the correct positions for interactions with α- and γ-phosphates. The second difference is the ~ 32° rotation of the apical domain (Fig. 4a). Since the apical domain is directly linked to the intermediate domain, it also moves toward AMPPNP in C-subunit. However, the domain has a rotational change in addition to the intermediate-domain flexure.

To visualize the effect of the additional rotation on ring closure, a hypothetical octamer model in the closed conformation was built where the apical domains do not rotate (Supplementary Fig. 11). The unrotated apical domains of the model collided during closure, indicating that the additional rotation relieves the steric clashes on ring closure.

The relatively small conformational change observed within single subunits upon ATP binding is not proportional to the concerted movement of interacting subunits on ring closure. The ATP-binding and consequent induced conformational change is amplified by inter-subunit articulation which is critical to elucidate the molecular mechanism of ring closure. The stem loop in one subunit forms a β-sheet with the C-terminal strand of an adjacent subunit (Fig. 2c). This inter-subunit β-sheet is maintained in the open and closed conformations although its position differs in the two conformational states (Figs. 2c and 3c). Notably, the β-sheet is the only inter-subunit contact in the open conformation, indicating that it is responsible for allosteric communication among subunits at the onset of ring closure.

To dissect the inter-subunit communication on ring closure, one octameric ring in the open conformation was superposed onto that of the closed conformation with equatorial domains as references (Fig. 4d). The superposition shows that each subunit rotates toward the 8-fold symmetry axis to close the chamber upon ATP-binding. This inward rotation is interlocked with the movement of the inter-subunit β-sheet. The C-terminal strand, forming a unit of the inter-subunit β-sheet, protrudes from one edge of the bottom of each subunit and supports the main body of each subunit. Hereafter, this stiff C-terminal element is referred to the C-terminal lever. As described above, ATP-binding induces the movement of the stem loop within a subunit, which exerts force on the C-terminal lever of the adjacent subunit through the inter-subunit β-sheet (Fig. 4d). As a result, the main body of the adjacent subunit rotates inward along the movement of the stem loop. In this way, the inter-subunit β-sheet transforms the ATP-induced movement of the stem loop in one subunit into the rotational movement of its adjacent subunit, closing the chamber.

The extreme termini of CPN subunits extending out from the equatorial domain are flexible and not resolved in structures. This is typical of both Group II and Group III CPNs. A Ch-CPN C-terminal deletion mutant missing the last 16 residues (Ch-CPN ΔC (506*)) was constructed to determine how this region affects complex assembly, thermostability, and ATPase activity. Native PAGE indicates that the Ch-CPN ΔC (506*) is still able to form an oligomeric complex (Supplementary Fig. 12a), but exhibits a ~ 15 °C decrease in optimum temperature and 27% of the basal ATPase activity of wild type (Supplementary Fig. 6b). These data support previous studies with Group II CPNs that demonstrate the extreme C-termini are involved in stabilizing inter-ring interactions in addition to the role the C-termini play in dynamic ATP-induced interactions resulting in the opening and closing of rings^26, 32^. These mutants will enable structure-function investigations beyond the mechanisms shown by structural analyses. Key studies on GroEL have emphasized the importance of CPN plasticity on the folding mechanism and revealed that the chambers can distort when bound to client proteins^33, 34^ and that the binding and release cycle of a CPN is a forceful process for both unfolding inappropriately folded substrates as well as promoting native folding^25^.

To observe inter-ring dynamics upon ring closure, we compared the inter-ring interface between the open and closed conformations (Fig. 4e). The interface between a pair of vertically contacting subunits is repeated around the ring and models the inter-ring articulation since the subunits do not form staggered interactions, as the GroEL complex does^34^. One remarkable and unique inter-ring contact that is preserved regardless of the conformational state is the interaction between Leu439 in one subunit and a groove formed by the backbone of residues 109–111 and the side chains of Lys109, Val111, Met411, and Tyr414 in the contacting subunit (Fig. 4e). The contact is conserved among widely divergent species with Group III CPNs (Supplementary Fig. 2, labeled “Socket”). The side chain of Leu439 fits into the groove, which resembles the ball-and-socket joints that connect two Lego pieces. Hereafter, the inter-ring contact between Leu439 and the groove is referred to the ball-and-socket joint (Fig. 4e).

**Fig. 6:**
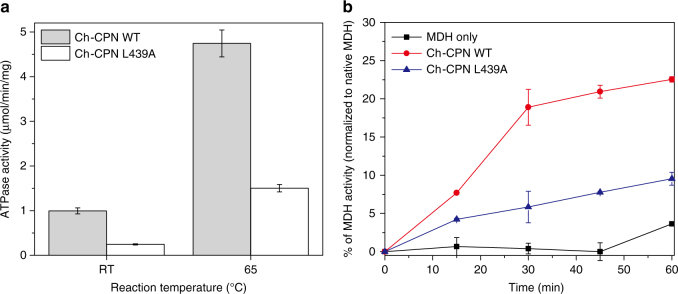
Comparison of ATPase and protein folding activity between Ch-CPN WT and the pivot joint mutant (L439A). Error bars are ± s.d. for triplicate experiments. **a** ATP hydrolysis rates of Ch-CPN compared with the L439A mutant. ATPase activities of Ch-CPN and the L439A mutant were determined after incubation for 5 min at the indicated temperature by using the malachite green assay. **b** Refolding of Malate Dehydrogenase (MDH) by Ch-CPN wild type and the L439A mutant. Denatured MDH was mixed with Ch-CPN and the L439A mutant at 42 °C, respectively. The specific activity of MDH prior to denaturation was the control used to determine 100%

We propose that the ball-and-socket joint acts as a pivot during opening and closing maneuvers. The inter-ring interface is divided into two parts (C-interface and O-interface) by the line connecting the two ball-and-socket joints (Fig. 4e). Inter-ring contacts occur at C- and O-interfaces in the closed and open conformations, respectively, indicating that the contact region changes from O-interface to C-interface on ring closure (Fig. 4e). For this change to occur, each subunit rotates toward the 8-fold symmetry axis with the line connecting two ball-and-socket joints as a pivot axis. It should be noted that this rotational movement is identical in each subunit. In order to test the action of this joint, the L439A mutant was constructed and its effects on the ATPase and protein refolding activities of the complex were tested. The L439A mutant subunits formed hexadecameric complexes and could be purified as hexadecameric chaperonins (Supplementary Fig. 12b). Both ATPase and refolding of MDH activities were depressed but not eliminated (Fig. 6), suggesting that the pivot joint with Leu as the pivot residue functions more efficiently than Ala in propagating the allosteric wave of articulation from one ring to the other.

In summary, an inter-subunit β-sheet formed by the stem loop and the C-terminal lever, and two ball-and-socket joints are key factors in communicating local changes in the nucleotide-binding site cooperatively throughout this hexadecameric protein machine to close the chambers. Nucleotide states are sensed by the stem loops in the inter-subunit β-sheets, and the concurrent movements of the β-sheets exert forces on the C-terminal lever. The operation of the levers rotates each subunit with respect to ball-and-socket pivot joints. On ring closure, the apical domains forming the built-in lid rotate as they swing together to avoid steric clashes (Supplementary Movie 1 and 2).

## Discussion

Here we present the structural and molecular characterization of monophyletic group of CPNs with potential protein folding roles in 195 diverse microbial species. Most of these are from extreme environments and situated on deep branches of the bacterial phylogenetic tree. Evolutionary characterization of the Group III CPN clade initially identified it as a very deep branch in the CPN evolutionary tree^23^, and the current phylogenetic analysis (Supplementary Fig. 1) confirms that, with many more sequences extracted from the burgeoning bacterial genome sequence collection, this conclusion still holds. Structural characterization of this prototype Group III CPN provides insights into the evolution of articulation mechanisms and ATP binding and hydrolysis that may provide insights into the origins of the efficient functioning of modern Group II CPNs.

The sequence, structure, and functional articulation of the lid and the nucleotide-sensing loop are remarkably different between Ch-CPN and TKS1-CPN (Fig. 5a). The most remarkable conformational differences are observed in the lids of closed chambers (Fig. 5b). The Ch-CPN lid (residues 223–263) contains two helices (α10 and α11) and a connecting loop between β7 and α10 whereas the TKS1-CPN lid is composed of one helix (H10), one β-strand, and a loop that corresponds to α10, α11, and the β7-α10 loop of Ch-CPN, respectively (Figs. 5c, d). A four-residue insertion at the region prior to α11 of Ch-CPN is associated with the transition of the helical region into a β-strand in TKS1-CPN (Fig. 5d). Due to the insertion-induced conformational transition, the N-terminal end of Η10 in TKS1-CPN is shifted upwards compared to the corresponding α10 in Ch-CPN and consequently, the tip of the TKS1-CPN lid is much wider than that of the Ch-CPN lid (Figs. 5a, c). In the closed form of Ch-CPN, eight α10 helices from each subunit are located side-by-side with no overlap at the center of the ring to close the chamber (Fig. 5b). In contrast, the β-strand and the loop of the lid in each subunit of TKS1-CPN overlap with adjacent lids to facilitate a tight closure of the chamber like Venetian blinds (Fig. 5b). Consequently, the radial angle of the lid domains of TKS1-CPN lid is offset ~ 45° compared with the Ch-CPN lid (Fig. 5c).

This structural incongruence of the lid domain suggests that Group II and Group III CPNs may rely on different client protein interaction mechanisms, and that the Ch*-*CPN chambers may be able to accommodate polypeptides of larger sizes due to the decreased separation between the cytosol and the interior of the folding chamber. Additionally, Group II and Group III CPNs may interact with different substrate-offloading co-chaperones. In the Archaea, a holdase type chaperone complex called prefoldin, interacts directly with the lid protrusion of Archaeal Group II CPNs via charged residues while delivering denatured substrates for refolding within the CPN chamber^35, 36^. Prefoldins, while occurring in all Archaea and Eukaryotes, are absent in bacteria, including *C. hydrogenoformans*. Eukaryotic CCT complexes have been reported to interact directly with Hsc70, a DnaK homolog^37^. The conserved genomic organization with Group III CPNs encoded within the DnaJ/K operon suggests that the observed structural variation in the lid may facilitate interaction with DnaK. Experiments to test this are in progress.

In Group II CPNs, several studies indicate that ATP binding to one ring transiently inhibits ATP binding to the other ring^38–41^. These studies have utilized multiple disciplines to determine structural plasticity including cryo-EM^27^ and show that nucleotide-bound crystal structures may present a biased view of the structural adaptability of CPNs. Due to negative allosteric inter-ring communication, only one ring is considered to be folding-active at a time and thus Group II CPNs are thought to function as so-called two-stroke motors^26^. Given that the built-in-lids in Group II CPNs initiate the negative allosteric communication between rings^41^, the unique lid structure of Group III CPNs implies that they may have different inter-ring communications. Remarkably, we observed very little cooperativity in the Group III complex, either by means of sub-saturating ATPase assays to generate a Hill plot (Supplementary Fig. 13), which gave a Hill coefficient of ~ 1, or by ATP saturation in the isothermal titration calorimeter, which showed that the complex could be fitted to a model equivalent to monomer binding (Supplementary Fig. 14 and Supplementary Table 2). Our results contrast with the archaeal Group II CPN open and closed structures shown by Huo et al.^42^ and more recent studies of Sahlan et al.^36^ supporting negative allostery.

The nucleotide-binding sites of Ch-CPN differ in structure and sequence from Group II CPNs (Figs. 5a, d). In the AMPPNP-bound Ch-CPN, the loop between α6 and α7 in the intermediate domain harbors Arg155 interacting with the γ-phosphate of AMPPNP whereas the loop, especially Arg155, has a different conformation in the ADP-bound Ch-CPN (Supplementary Fig. 5). The arginine residue is completely conserved across all known Group III CPN sequences and the mutation of the residue to acidic amino acids or alanine led to a severe defect in ATPase activity (Supplementary Fig. 6). This, together with the retained ATPase activity of the R155K mutant, establishes the importance of a positively charged residue interacting with the γ-phosphate (Supplementary Fig. 5). By comparison, in Group II CPNs, the loop corresponding to the α6-α7 loop of Ch-CPN is prolonged by six residues and covers the ATP-binding site (Fig. 5d). This nucleotide-sensing loop harbors a conserved lysine residue interacting with the γ-phosphate of ATP and seems to be implicated in the regulation of the protein folding cycle by changing its conformation depending on bound nucleotides^43^.

The disparity in the nucleotide-sensing loop between Group II and Group III is associated with their different local conformational changes on ATP binding. The movements of the whole intermediate domain and the stem loop in Ch-CPN was observed depending on nucleotide identity (Figs. 4a–c) whereas in Group II CPNs only the prolonged nucleotide-sensing loop undergoes rearrangements in several nucleotide-bound states without the movement of the whole intermediate domain and the stem loop^43^. We propose that this disparity in local conformational change on nucleotide binding likely affects the timing of ring closure. The closed structure of the AMPPNP-bound Ch-CPN and the open structure of the ADP-bound Ch-CPN indicate ring closing upon ATP binding. According to several biochemical and structural studies^41, 44^, however, ATP binding alone is not sufficient to trigger ring closure and Group II CPNs are actually closed during ATP hydrolysis. It would appear that in Group II CPNs the conformational transition of ATP binding is cushioned by the structural rearrangement of the long nucleotide-sensing loop and subsequent ATP hydrolysis is necessary to energize larger conformational changes for ring closing.

In conclusion, the deep phylogenetic separation between the Group III clade and the CPNs in the Group II branches, in conjunction with our findings that the Ch-CPN is mechanistically distinct from both Group I and Group II CPNs, supports our hypothesis that it is related to the Last Common Ancestor of modern CPNs. The alternative possibility is that a lateral gene transfer of a Group II CPN from an Archaeon to a member of the Firmicute phylum took place early during the divergence of Group II CPNs. However, the discovery of non-canonical archaeal CPN genes similar to Ch*-*CPN in the very deep branch of the Thaumarchaeota (see Supplementary Fig. 1) also supports our contention that a CPN similar to Group III CPNs gave rise to Group II CPNs. A Group I CPN may have arisen by the excision of the lid region of a Group III CPN to form the GroES genes that occur in all bacteria.

## Methods

### Protein preparation

Recombinant proteins for use in in vitro assays were expressed using auto-induction with BL21 *Escherichia coli* (Novagen) transformed with a modified pET24b( + ) vector (Novagen) containing a *tac* promoter cloned at *Bgl*II and *Xba*I restriction sites^45, 46^. Ch-CPN gene was inserted at *Nde*I and *Xho*I restriction sites. Cells were collected by centrifugation at 5,000 × *g* for 10 min at room temperature, then resuspended (5% wt vol^−1^) in 50 mM NaH_2_PO_4_ (pH 8.0) and 300 mM NaCl in the presence of 1 mM phenylmethylsulfonyl fluoride followed by lysis via French Press. Proteins used in ATPase assays were initially resuspended and lysed in HEPES buffer (50 mM HEPES (pH 8.0) and 100 mM NaCl) to eliminate background phosphate. The lysate was centrifuged at 20,000 × *g* for 20 min at room temperature. The supernatant was heated at 50 °C for 30 min followed by additional centrifugation at 20,000 × *g* for 20 min to remove aggregation. Heat-treated and clarified supernatant was loaded onto anion exchange High Q column (Bio-Rad) and eluted in a linear gradient up to 1 M NaCl. Protein concentrations were determined by Coomassie Plus (Bradford) reagent (Pierce) at 595 nm. Site-directed mutagenesis was performed following the QuikChange II protocol (Agilent Technologies). The primer sequences used for constructing expression plasmids are described in Supplementary Table 3.

### Crystallography

The full-length gene of Ch-CPN WT coding for a 521-residue polypeptide was inserted downstream of the tac promoter of the expression plasmid pTAC24b + (courtesy of Dr. Zeev Pancer) using *Nde*I and *Xho*I (NEB Inc.)^44^. The plasmid DNA was transformed into the *E. coli* BL21 (DE3) (Novagen). The transformed cells were grown to an OD_600_ of 0.5 at 37 °C in Luria-Bertani media (Merck) with 50 mg ml^−1^ kanamycin (Duchefa) and the overexpression of Ch-CPN was induced by the addition of 1 mM isopropyl-1-thio-β-D-galactopyranoside (Duchefa)^44^. Cells were collected and disrupted by sonication after 16 h induction at 20 °C. After centrifugation 20,000 × *g* for 60 min, the supernatant fraction was loaded onto a Q-Sepharose column (Amersham Pharmacia Biotech) and then eluted fractions containing Ch-CPN were applied to a t-Butyl HIC column (Bio-Rad)^44^. Fractions containing Ch-CPN were finally loaded on to a Superdex 200 HR 16/60 size exclusion column (GE Healthcare) equilibrated with a 20 mM NaH_2_PO_4_ (pH 7.0) buffer^44^. The purified recombinant Ch-CPN protein was concentrated to  ~ 17 mg ml^−1^ for crystallization^44^. To make the Ch-CPN/AMPPNP and Ch-CPN/ADP complexes, 3 mM AMPPNP (Roche) and ADP (Sigma) were added to the protein solution, respectively^44^.

Crystals were obtained at 22 °C by the batch crystallization method. The mother liquor consisting of 20 mM MgCl_2_, 100 mM HEPES: NaOH (pH 7.5), and 22% Polyacrylic Acid 5,100, and 4% 1,1,1,3,3,3-Hexafluoro-2-propanol was used for crystallization of the Ch-CPN/AMPPNP complex^44^. Crystals of the Ch-CPN/ADP complex were grown in a solution containing 200 mM NH_4_Cl, 10 mM MgCl_2_, 50 mM HEPES sodium (pH 7.0), 5% w/v Polyethylene glycol PEG 8,000, and 3% 1,8- Diaminooctane^44, 47^. For data collection at −173 °C using crystals flash-cooled by a Cryostream Cooler (Table 1), 20% glycerol was added to the same mother liquors as a cryoprotectant. After molecular replacement with a Group II chaperonin from *Methanococcus maripaludis* (PDB code: 3KFE) as a search model, initial models were manually modified by using Coot^48^ and subsequently refined by using Phenix^49^. Several rounds of manual remodeling and refinement produced the final models of the Ch-CPN/AMPPNP and Ch-CPN/ADP complexes that were refined to 3.0 and 4.0 Å resolutions, respectively (Table 1). The final models of four Ch-CPN/AMPPNP complexes in the asymmetric unit contain residues 10–502, four magnesium ions, and four AMPPNP molecules. Residues 1–9 and 503–521 are completely disordered. The final models of four Ch-CPN/ADP complexes in the asymmetric unit contain residues 9–502 and four ADP molecules. Residues 1–8 and 503–521 are completely disordered. The Ramachandran plots of the final models indicate that 96.69% (Ch-CPN/AMPPNP complexes) and 93.24% (Ch-CPN/ADP complexes) of the non-glycine residues are in the most favored regions and the remaining 2.9% (Ch-CPN/AMPPNP complexes) and 6.35% (Ch-CPN/ADP complexes) residues are in allowed regions.

### ATPase activity

Liberated P_i_ resulting from ATP hydrolysis was measured using the Malachite Green assay at 620 nm^49^. Standard reaction mixture contained 50 mM HEPES (pH 7.5), 50 mM KCl, 20 mM MgCl_2_, 0.1 mM ATP, and Ch-CPN (0.06–0.1 mg ml^−1^). The mixture was incubated at the indicated temperature for 5 min or 60 min. The filtered malachite green dye (136 μl) was put into the reaction mixture (8 μl). Subsequently, 34% citric acid (16 μl) was added to prevent any further color development after 1 min.

### Malate dehydrogenase refolding assay

Porcine heart malate dehydrogenase (MDH) (Sigma-Aldrich) was denatured in denaturation buffer (25 mM HEPES (pH 7.2), 300 mM NaCl, 1 mM MgCl_2_, 4 M Guanidine-HCl, and 5 mM Dithiothreitol) at 37 °C for 1 h. Denatured MDH was diluted 100-fold into refolding buffer (25 mM HEPES (pH 7.2), 300 mM NaCl, 1 mM MgCl_2_, 1 mM ATP, and 0.5 M ammonium sulfate) in the presence Ch-CPN WT or L439A. The concentration of denatured MDH was 375 nM and the ratio of Ch-CPN and to denatured MDH was ~ 1:10. The reactions were incubated at 42 °C for 1 h. MDH activity was measured in MDH buffer (50 mM Tris-HCl (pH 8.0), 0.2 mM NADH, and 0.5 mM oxaloacetate) at 25 °C. The final concentration of MDH was 37.5 nM in MDH assay. The time dependent oxidation of β-NADH was monitored at 340 nm.

### Proteinase K sensitivity assay

Purified wild type Ch-CPN (0.5 mg ml^−1^) was pre-incubated with 10 mM ADP, ATP, or AMPPNP in proteinase K buffer (50 mM Tris-HCl (pH 7.4), 150 mM NaCl, 25 mM MgCl_2_) for 15 min at 65 °C. After addition of proteinase K (20 mg ml^−1^) and further incubation for 10 min at room temperature, phenylmethylsulfonyl fluoride was added at a final concentration of 5 mM to inhibit proteinase K activity. The reaction mixtures analyzed by SDS-PAGE.

### Isothermal titration calorimetry

Measurements of the ATP binding were made in a Nano ITC 1 ml Volume (New Castle, DE, TA Instruments). ATP concentrations were measured spectrophotometrically at 259 nm with an extinction coefficient of 15,400 M^−1^ cm^−1^. All experiments were carried out at 55 °C in ITC buffer (20 mM Tris-HCl (pH 7.6), 20 mM MgCl_2_, and 10 mM KCl). Prior to use, all solutions were degassed under vacuum to eliminate air bubbles. Titration experiments were performed by successive 5 μl injections of 200 μM ATP solution into proteins, both prepared in ITC buffer (0.43 μM for the hexadecamer). The interval between injections was 300 s, and stirring at 300 r.p.m. ensured a good mixing. Binding isotherms were corrected by subtracting the ligand dilution isotherms, determined by titrating ATP solution into the buffer. Data analysis was carried out using MicroCal Origin 7.0.

### Data analyses

Data were expressed as means ± s.d. from three independent experiments. Statistical analysis was performed using Student’s *t*-test. Differences were considered statistically significant with *P* < 0.04.

### Data availability

The atomic coordinates and structure factors have been deposited in the Protein Data Bank, www.pdb.org (PDB ID codes 5X9V (closed form) and 5X9U (open form)). Other supporting data are available from the corresponding authors upon reasonable request.

## Electronic supplementary material

Supplementary Information

Description of Additional Supplementary Files

Supplementary Movie 1

Supplementary Movie 2
