# Supplementary material for: Structural and mechanistic characterization of an archaeal-like chaperonin from a thermophilic bacterium
Source: Nat Commun. 2017 Oct 10;8:827. doi: 10.1038/s41467-017-00980-z (PMC5635000; doi:10.1038/s41467-017-00980-z)
Supplement: Supplementary file 1 — Supplementary Information [file 41467_2017_980_MOESM1_ESM.pdf]

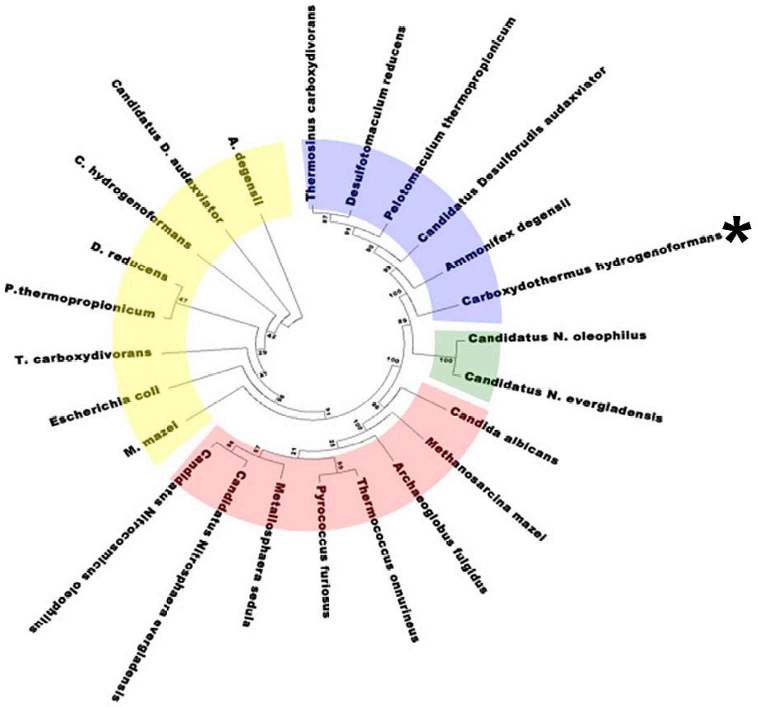

Supplementary Figure 1. Maximum Parsimony analysis of taxa with Group III CPN homologs. Main figure: Radial view. *Carboxydothermus hydrogenoformans*, the source of the CPN used in this study, is marked with an asterisk (*).

Colour coding:

Group I, Yellow,

Group II, Orange,

Group III, Blue. Group III are representatives from 185 available homologs.

The Green shading indicates a new Thaumarchaeota branch, possible predating Group III. The evolutionary history was inferred using the Maximum Parsimony method. The MP tree was obtained using the Subtree-Pruning-Regrafting (SPR) algorithm with search level 1 in which the initial trees were obtained by the random addition of sequences (10 replicates). The analysis included polypeptide sequences. Gaps were eliminated. There were a total of 437 positions in the final dataset. Evolutionary analyses were conducted in MEGA7[1]. The species and gene accession number for each of the CPN sequences are described in Supplementary Table 1.

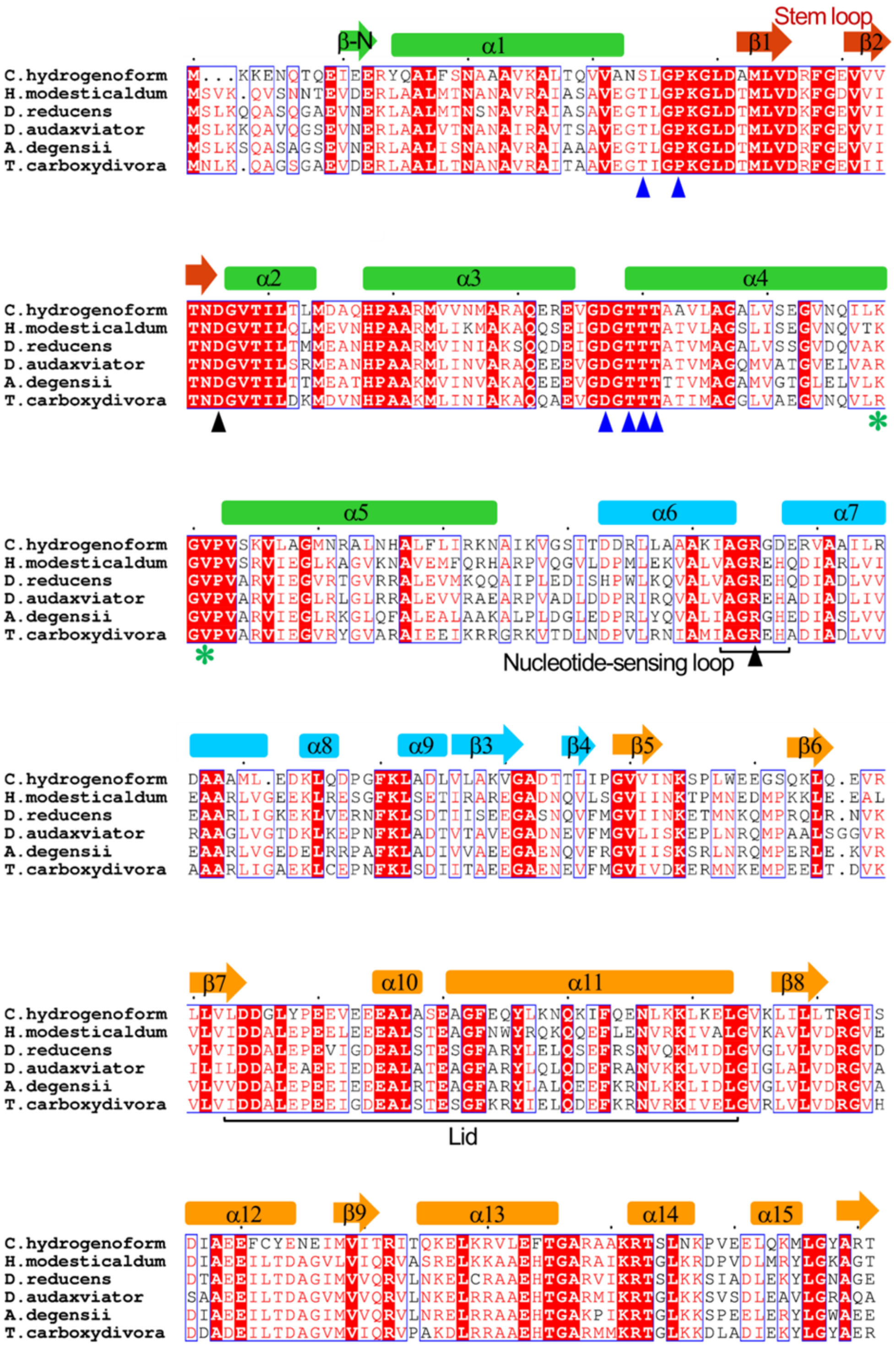
Stem loop
β-N
α1
β1
β2
C.hydrogenoform
H.modesticaldum
D.reducens
D.audaxviator
A.degensii
T.carboxydivora
α2
α3
α4
α5
α6
α7
Nucleotide-sensing loop
α8
α9
β3
β4
β5
β6
β7
α10
α11
β8
Lid
α12
β9
α13
α14
α15

Continue →

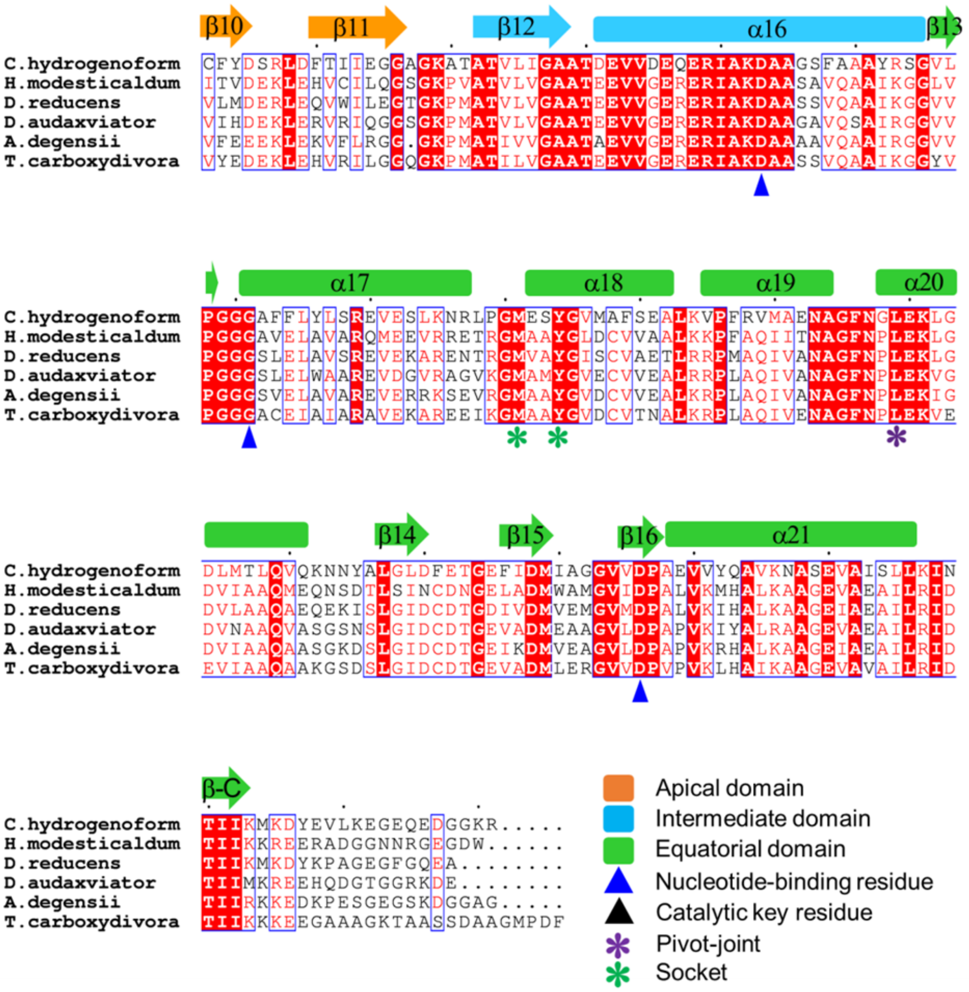

Supplementary Figure 2. Structure-based sequence alignment of Group III CPNs.

Alignment of Group III CPN sequences is generated by using ESPript. The secondary structure assignments correspond to Ch-CPN. The identical residues are highlighted with red background and the conserved residues are indicated by red letters and blue boxes. Nucleotide-binding residues and catalytic key residues are indicated by blue and black triangles, respectively. Pivot-joint and socket residues are marked by purple and green asterisks, respectively. Two terminal β-strands only observed in the closed form are labeled as β-N and β-C, respectively. Expasy numbers of the aligned Group III CPN are as follow: *Carboxydothermus hydrogenoformans* (Q3AF10), *Heliobacterium modesticaldum* (B0TAD5), *Desulfotomaculum reducens* (A4J7F5), *Desulforudis audaxviator* (B1I6A1), *Ammonifex degensii* (C9RBX6), and *Thermosinus carboxydivorans* (A1HR08).

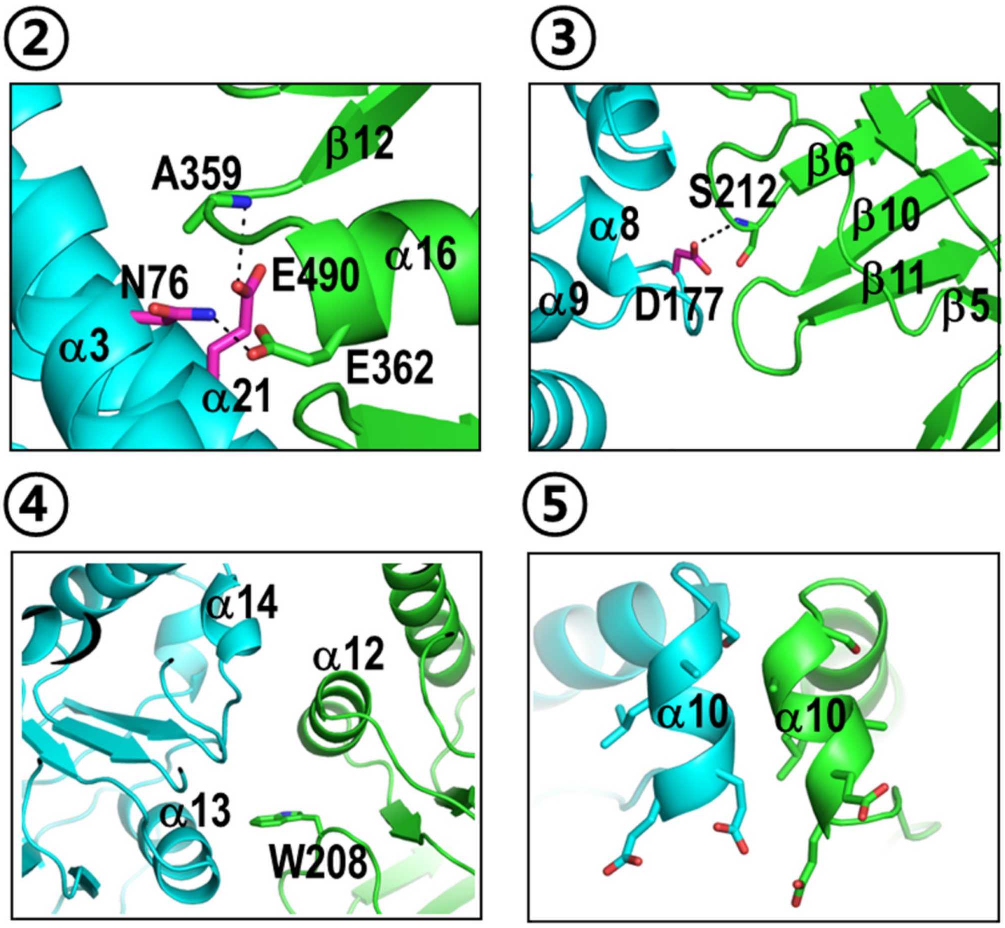

Supplementary Figure 3. Close-up views of interaction sites in closed conformation of Ch-CPN. ② is the lower contact region formed by equatorial domains and others (from ③ to ⑤) are the interactions mediated by apical and intermediate domains. Interacting residues and hydrogen bonds are represented in sticks and dashed lines, respectively.

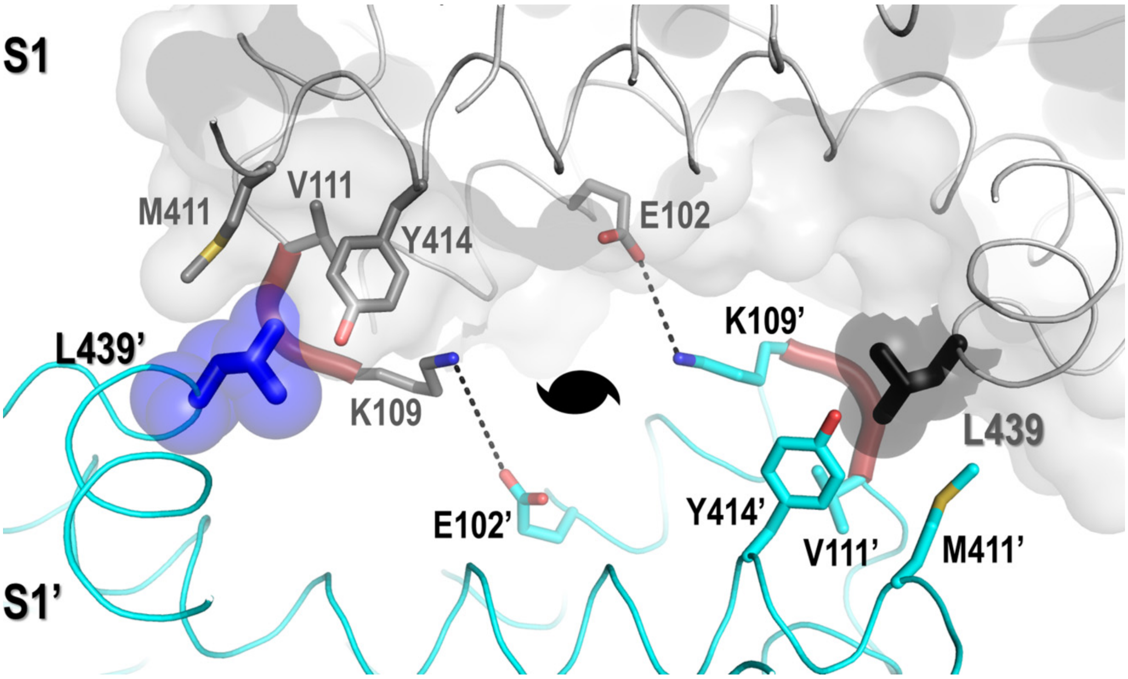

Supplementary Figure 4. Close-up view of the S1-S1' interface in the closed conformation. The backbones of residues 109-111 and important residues of the S1 (gray) and S1' (cyan) interface are represented in cartoons (red) and sticks, respectively. Dotted black lines indicate polar interactions.

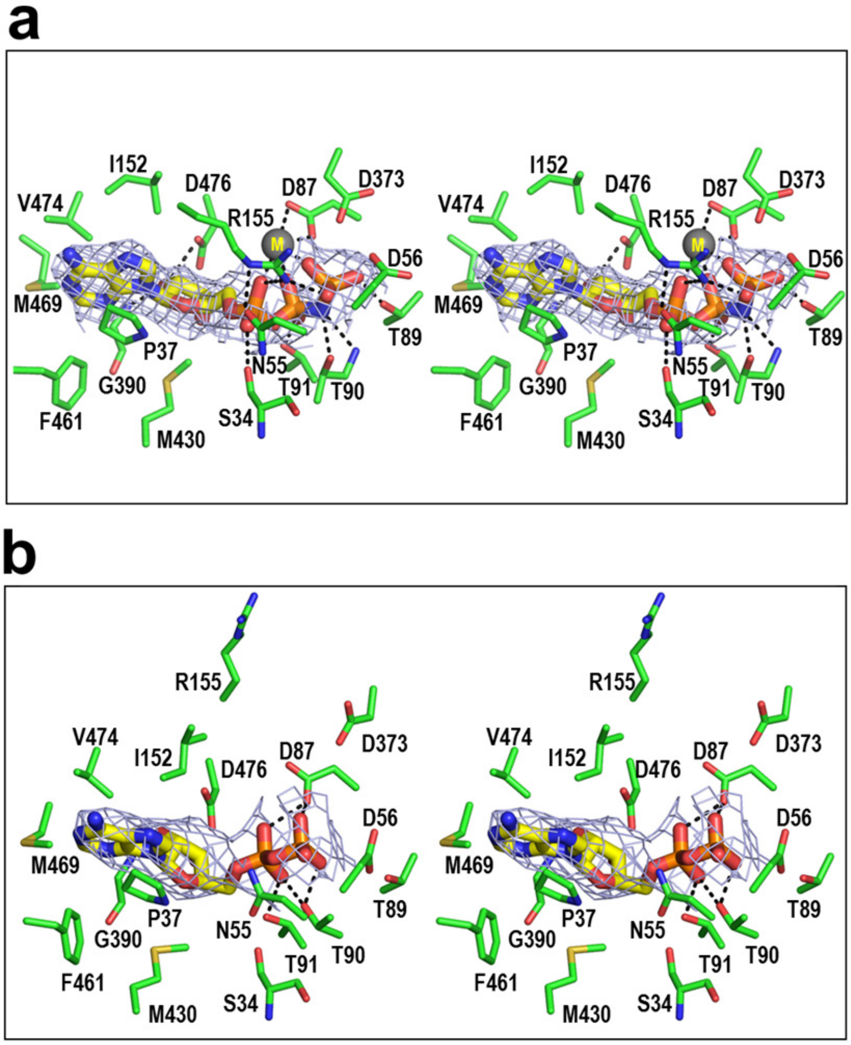

Supplementary Figure 5. Stereo views of nucleotide-binding sites of the AMPPNP-bound (a) and ADP-bound (b) Ch-CPNs. AMPPNP/ADP and interacting residues are shown as yellow and green sticks, respectively. A magnesium ion symbolized by M and hydrogen bonds are indicated by a gray sphere and dashed lines, respectively. Simulated annealing $2F_o$-F_c omit maps contoured at 1.0 σ for ADP and AMPPNP are shown: at a refinement stage with an R value of 25.8% (the AMPPNP-bound structure) or 32.2% (the ADP-bound structure), each structure was disturbed at 5,000 K with the omission of AMPPNP or ADP and then electron density maps were calculated with the same omission.

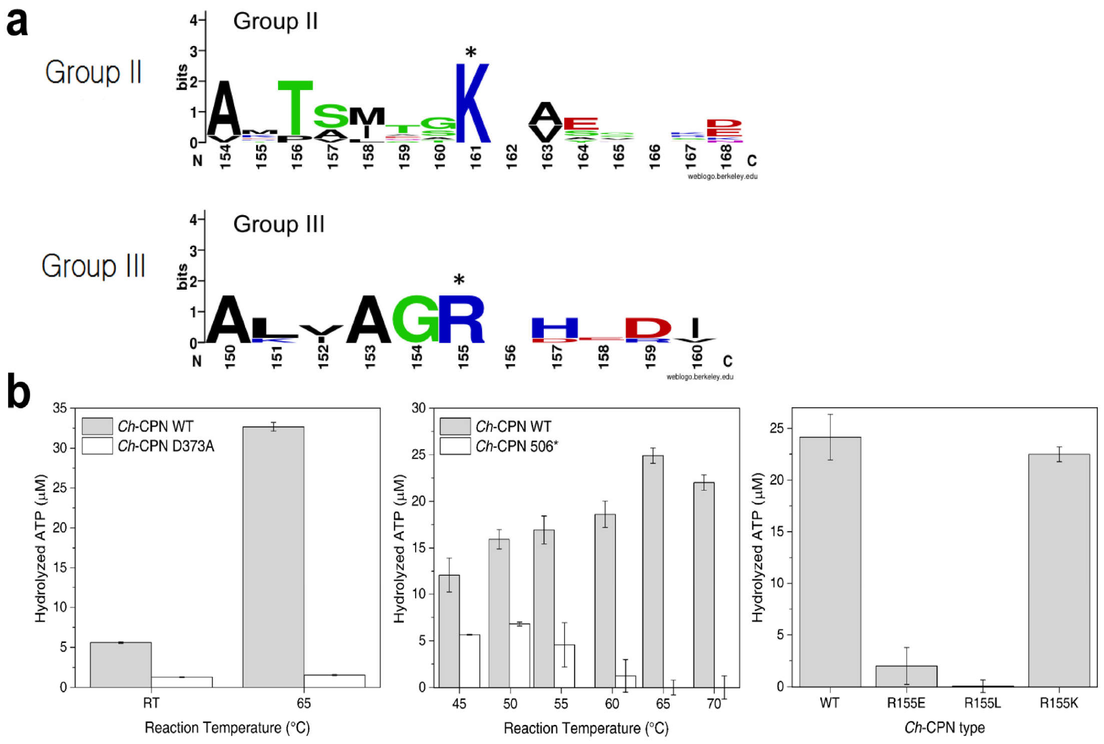

Supplementary Figure 6. Sequence and biochemical analysis on ATPase activity of Ch-CPN. (**a**) Highly conserved ATP γ-phosphate interacting residues of Group II and Group III chaperonins (asterisks). Top: Logo of 26 Group II chaperonin sequences using representatives from all 8 distinct eukaryotic subunits. Bottom: Logo of 79 Group III chaperonin sequences extracted from NCBI database based on Ch-CPN BLASTp results[2]. Arg155 is strictly conserved in Group III CPN corresponding to Lys in Group II. Amino acid logos were created by using Weblogo[3,4] with ClustalW generated alignments as input. The residues in top Logo and bottom Logo are numbered according to the numbering of *Methanococcus marapaludis* CPN and Ch-CPN, respectively. (**b**) ATPase activities of Ch-CPN wild type (WT) compared with ATP-hydrolysis (D373A; left), nucleotide-binding (R155E, R155L, and R155K; middle), and C-terminal deletion mutants (right). The ATP hydrolyzing activities were determined after incubation for 60 min at the indicated temperature. Error bars are ±standard deviation for triplicate experiments.

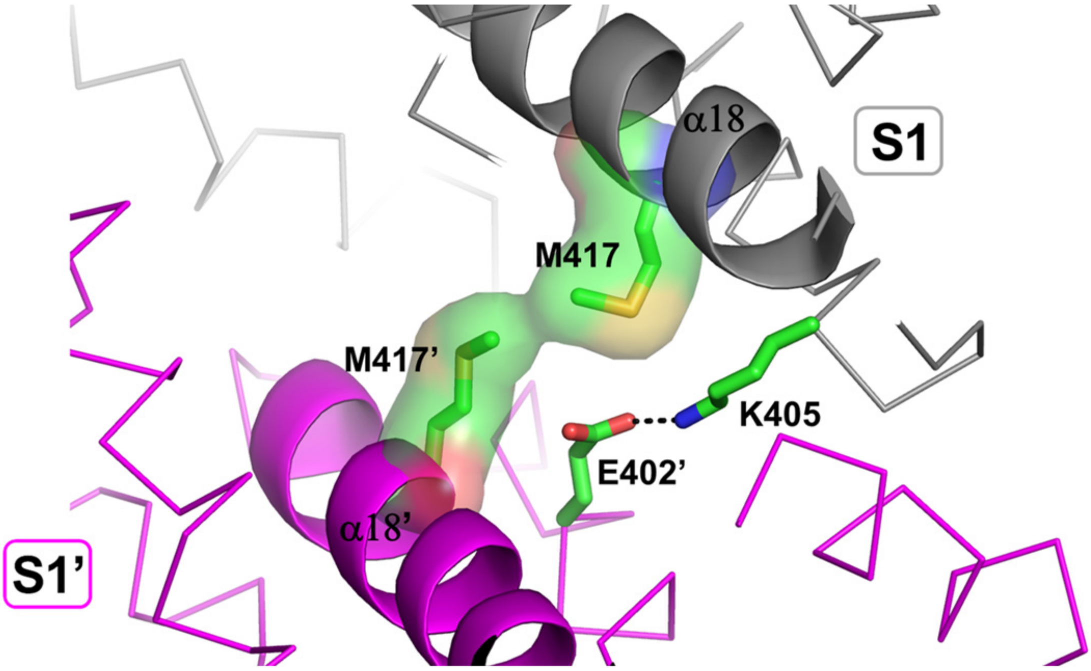

Supplementary Figure 7. Close-up view of the S1-S1' interface in the open conformation. Interacting residues and hydrophobic contacts are represented in sticks and surface, respectively. A dotted black line indicates a polar interaction.

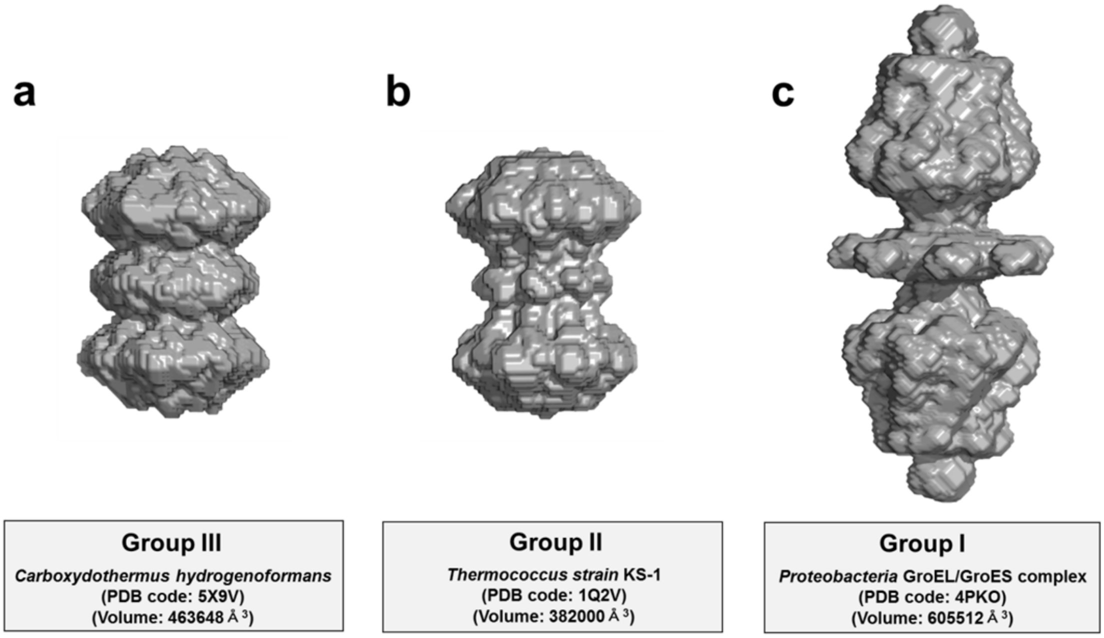

Supplementary Figure 8. Comparison of volumes of Group I, II, and III Chaperonins. Volumes of the internal chamber of the (**a**) Group III chaperonin (left), (**b**) Group II chaperonin (middle), and (**c**) Group I chaperonin (right) were calculated by using the 3V: Voss Volume Voxelator server[5]. The volume images are drawn to the same scale.

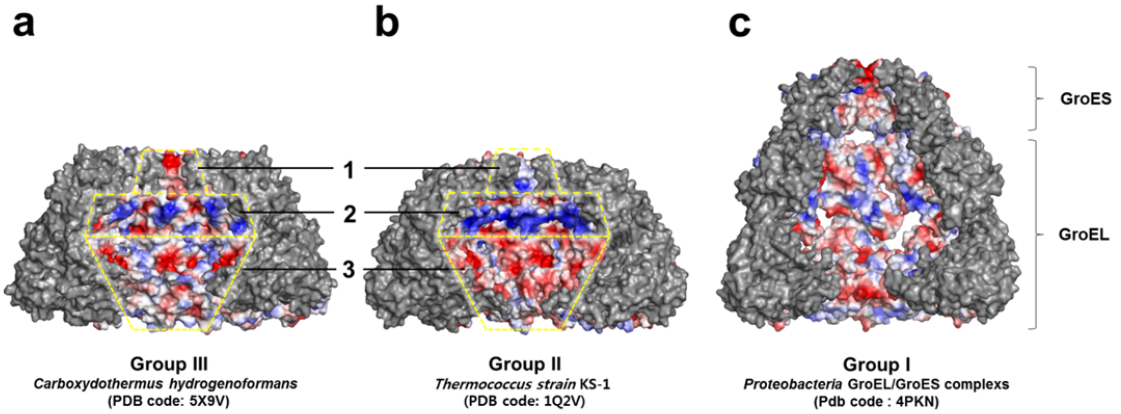

Supplementary Figure 9. Comparison of electrostatic surface of the folding chamber. Electrostatic potentials of the (a) Group III (b) Group II, and (c) Group I chaperonins calculated by PyMOL[6] are shown in blue for positively charged area and red for negatively charged area, respectively. The first, second, and third layers are indicated by 1, 2, and 3, respectively (yellow dotted boxes).

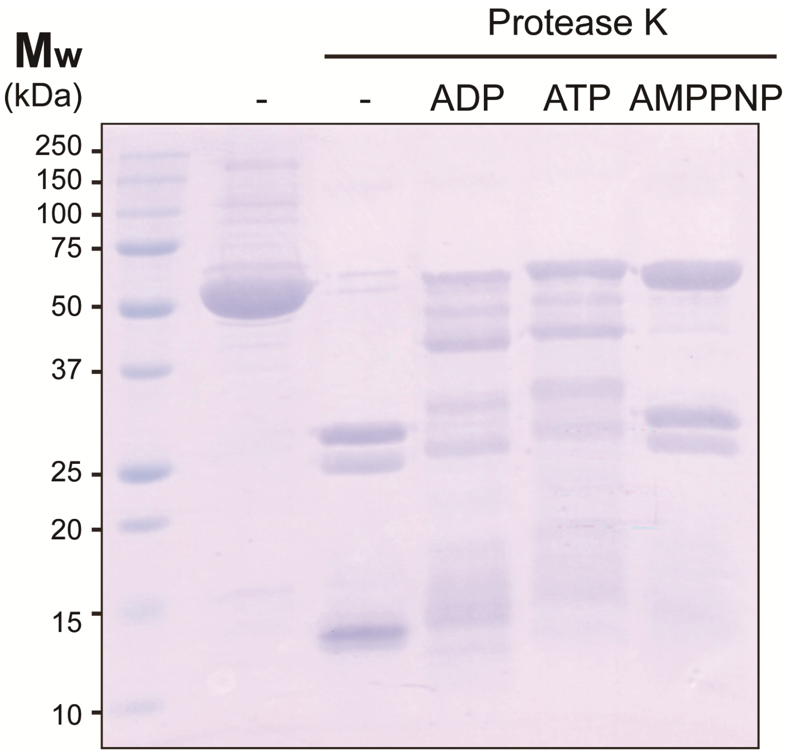

Supplementary Figure 10. Proteolytic analysis of Ch-CPN with proteinase K. Ch-CPN WT was preincubated with or without 10 mM of the different nucleotides (ADP, ATP, and AMPPNP) at 65 ^{o}C for 15 min. After incubation, proteinase K was added to the solution. The reaction mixtures were analyzed by SDS-PAGE.

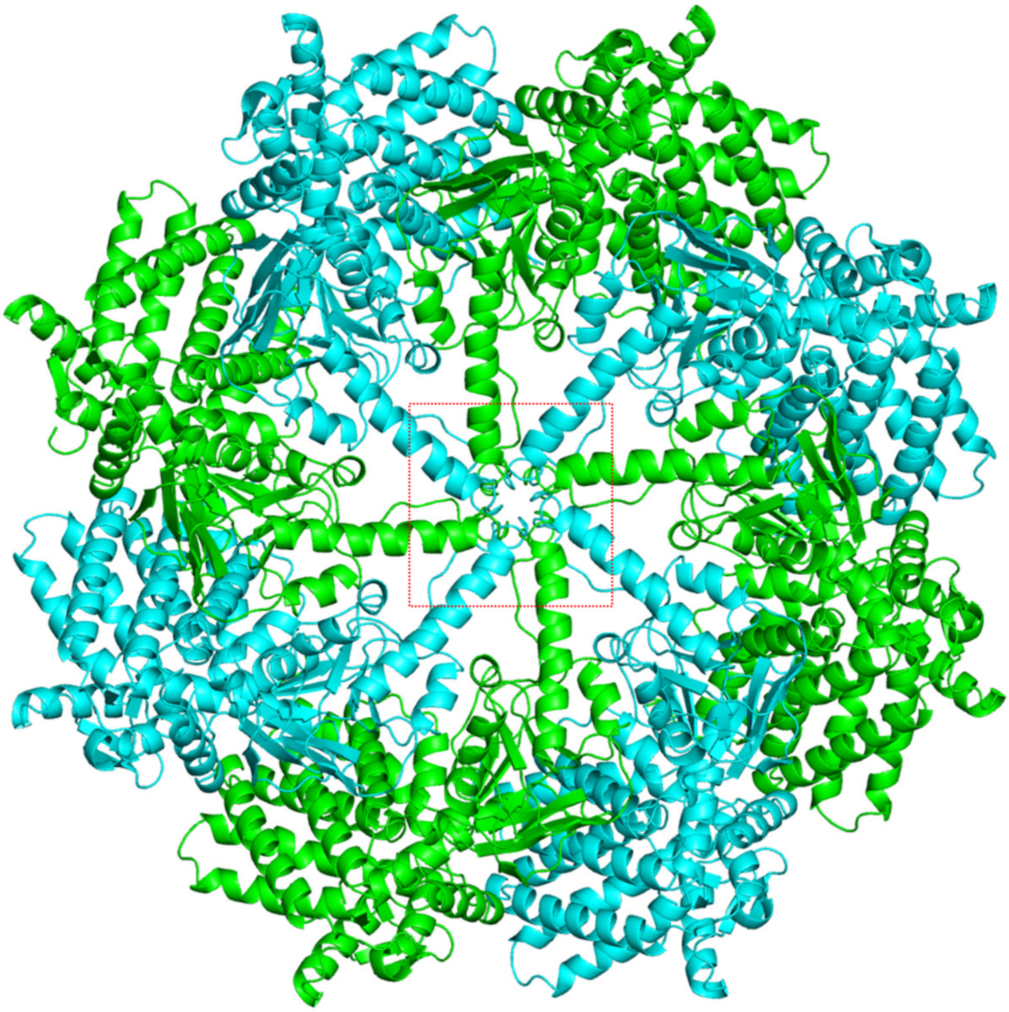

Supplementary Figure 11. Hypothetical octamer model in the closed conformation. Red dotted box indicates steric clashes.

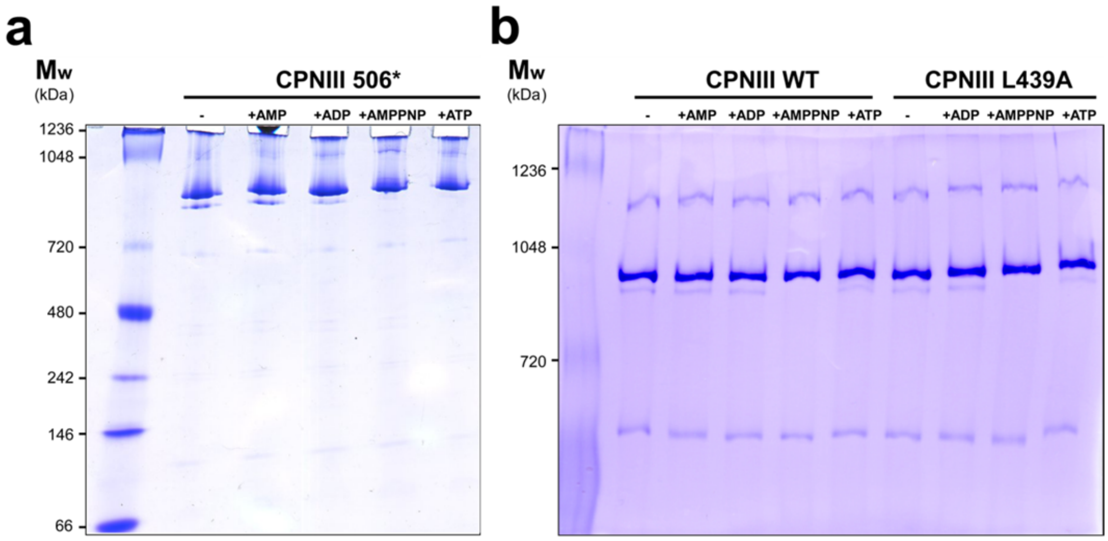

Supplementary Figure 12. Native PAGE of Ch-CPN (a) 506*, (b) WT, and L439A mutants in the presence of AMP, ADP, ATP, or AMPPNP. Ch-CPN WT, 506*, and L439A were incubated with or without 2 mM of the different nucleotides (ADP, ATP, and AMPPNP) at 65 ^{o}C for 10 min. After incubation, Ch-CPN or /nucleotide complexes analyzed by (a) 4-16% gradient or (b) 4% native PAGE.

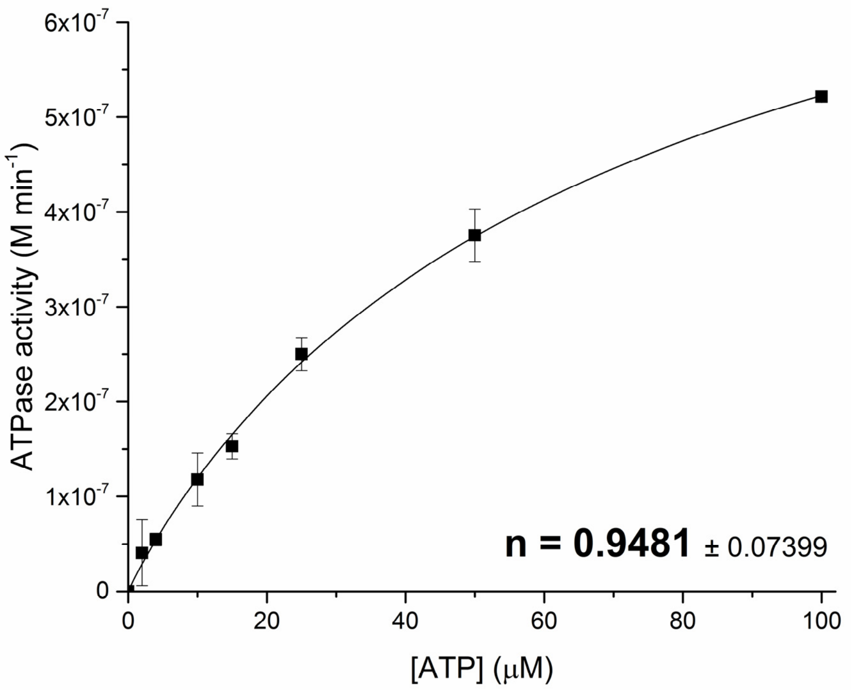

Supplementary Figure 13. Initial rates of ATPase activity of Ch-CPN WT as a function of ATP concentration. The continuous curve was obtained by fitting the data to the Hill equation using Origin software (OriginPro 2015, USA). The data represents the average of the three independent experiments. Error bars indicate standard deviations.

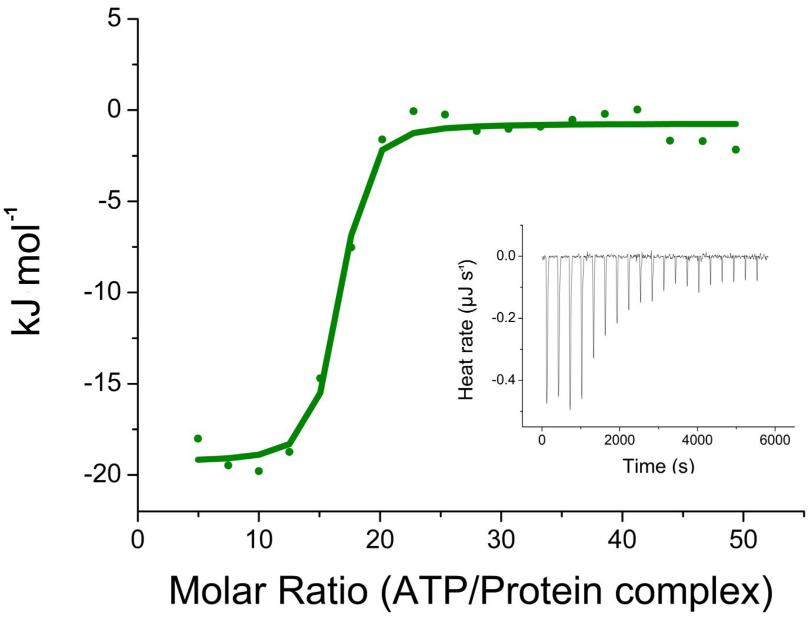

Supplementary Figure 14. Isothermal titration calorimetry (ITC) anlaysis of interaction between ATP and the D373A mutant of Ch-CPN. Measurements of ATP binding to Ch-CPN were made using a Nano ITC (1 ml cell volume) (TA Instruments, Newcastle, DE) using the D373A mutant of Ch-CPN. Experiments were carried out at 55°C in 20 mM Tris-HCl, 20 mM $MgCl_2$ and 10 mM KCl (pH 7.6). Titration experiments were performed by successive 5 µL injections of freshly prepared 200 µM ATP solution into proteins (0.43 µM for the hexadecamer). The interval between injections was 300 s, with stirring at 300 rpm. Binding isotherms were corrected by subtracting the ligand dilution isotherms, determined by titrating ATP solution into buffer alone. The observed binding isotherms were fitted to the "one set of sites" model (green line). In this model, the putative chaperonin has 16 identical nucleotide binding sites, which are independent of each other and have a uniform binding constant, K_a, and enthalpy change, ΔH. Data analysis was carried out using MicroCal Origin 7.0.

Inset: Thermal power (µJ sec^{-1}) as a function of time (sec) for the ITC binding experiment during titration of 20 individual injections.

Supplementary Table 1. Species name and accession number of 24 CPNs for Maximum Parsimony analysis

Genus	Species	Gene_ID	CPN Group
Ammonifex	*degensii*	Adeg_0161	I
Ammonifex	*degensii*	Adeg_0605	III
Archaeoglobus	*fulgidus*	XD48_0376	II
Candida	*albicans*	MG5_00007	II
Candidatus Desulforudis	*audaxviator*	Daud_2007	I
Candidatus Desulforudis	*audaxviator*	Daud_2059	III
Candidatus Nitrocosmicus	*oleophilus*	NMY3_00516	?
Candidatus Nitrocosmicus	*oleophilus*	NMY3_03297	II
Candidatus Nitrocosmicus	*evergladensis*	NTE_02889	?
Candidatus Nitrosphaera	*evergladensis*	NTE_00525	II
Carboxydothermus	*hydrogenoformans*	CHY_0807	III
Carboxydothermus	*hydrogenoformans*	CHY_0413	I
Desulfotomaculum	*reducens*	Dred_2873	I
Desulfotomaculum	*reducens*	Dred_2498	III
Escherichia	*coli*	-	I
Methanosarcina	*mazei*	DU84_05305	I
Methanosarcina	*mazei*	DU81_00490	II
Metallosphaera	*sedula*	HA72_2264	II
Pelotomaculum	*thermopropionicum*	PTH_2605	I
Pelotomaculum	*thermopropionicum*	PTH_0876	III
Pyrococcus	*furiosus*	PF1974	II
Thermosinus	*carboxydivorans*	TcarDRAFT_0384	I
Thermosinus	*carboxydivorans*	TcarDRAFT_1251	III
Thermococcus	*onnurineus*	TON_0707	II

?: a new Thaumarchaeota branch, possible predating Group III.

Supplementary Table 2. Thermodynamic parameters of Ch-CPN D373A determined by fitting the ITC data

Protein	N	K_d (M)	K_a (M^{-1})	ΔH (kJ mol^{-1})	ΔS (J mol^{-1} K^{-1})
**Ch-CPN D373A**	16	3×10^{-8}	3×10^{7}	-18	87.85

Supplementary Table 3. Primers used for constructing expression plasmids

Protein	Primer sequences	
**Ch-CPN WT**	**Forward**	AAAAAACATATGAAAAAAGAAAACCAAA
	**Reverse**	TTTTTTCTCGAGTTATCTTTTTCCTCCATC
**Ch-CPN R155E**	**Forward**	CGGCCAAAATTGCCGGAGAGGGTGATGAAAGGGTTGC
	**Reverse**	GCAACCCTTTCATCACCCTCTCCGGCAATTTTGGCCG
**Ch-CPN R155K**	**Forward**	CGGCCAAAATTGCCGGAAAGGGTGATGAAAGGGTTGC
	**Reverse**	GCAACCCTTTCATCACCCTTTCCGGCAATTTTGGCCG
**Ch-CPN R155L**	**Forward**	CGGCCAAAATTGCCGGACTGGGTGATGAAAGGGTTGC
	**Reverse**	GCAACCCTTTCATCACCCAGTCCGGCAATTTTGGCCG
**Ch-CPN D373A**	**Forward**	CAGGAAAGAATTGCTAAAGCTGCGGCCGGAAGTTTTGCCG
	**Reverse**	CGGCAAAACTTCCGGCCGCAGCTTTAGCAATTCTTTCCTG
**Ch-CPN L439A**	**Forward**	GCCGGTTTTAACGGGGCGGAAAAGTTGGGGGAC
	**Reverse**	GTCCCCCAACTTTTCCGCCCCGTTAAAACCGGC
**Ch-CPN 506***	**Forward**	ACCATAATTAAAATGAAATAATAGGAAGTTTTAAAGGAAGGGGAGCAG
	**Reverse**	CTGCTCCCCTTCCTTTAAAACTTCCTATTATTTCATTTTAATTATGGT

References for Supplementary information

1. Kumar S., Stecher G., and Tamura K. MEGA7: Molecular Evolutionary Genetics Analysis version 7.0 for bigger datasets. *Mol. Biol. Evol.* **33**:1870-1874. (2016).

2. Altschul SF, Gish W, Miller W, Myers EW, Lipman DJ. Basic local alignment search tool. *J. Mol. Biol.* 1990 Oct 5;**215**(3):403-410. (1990).

3. Schneider TD, Stephens RM. Sequence logos: A new way to display consensus sequences. *Nuc. Acids Res.***18**:6097–6100. (1990).

4. Crooks GE, Hon G, Chandonia JM, Brenner SE. WebLogo: a sequence logo generator. *Genome Res.* **14**(6):1188-1190. (2004).

5. Voss NR, Gerstein M. 3V: cavity, channel and cleft volume calculator and extractor. *Nucleic Acids Res.* 38, W555-W562 (2010).

6. Schrodinger, LLC. The PyMOL Molecular Graphics System, Version 1.8. (2015).
