# Supplementary material for: Structural and mechanistic characterization of an archaeal-like chaperonin from a thermophilic bacterium
Source: Nat Commun. 2017 Oct 10;8:827. doi: 10.1038/s41467-017-00980-z (PMC5635000; doi:10.1038/s41467-017-00980-z)
Supplement: Supplementary file 2 — Description of Additional Supplementary Files [file 41467_2017_980_MOESM2_ESM.pdf]

Description of Supplementary Files

File name: Supplementary Movie 1
Description: The side view of ring closure dynamics

File name: Supplementary Movie 2
Description: The top view of ring closure
